# Levels of circulating tumor DNA correlate with tumor volume in gastro‐intestinal stromal tumors: an exploratory long‐term follow‐up study

**DOI:** 10.1002/1878-0261.13644

**Published:** 2024-05-24

**Authors:** Roos F. Bleckman, Charlotte M. S. C. Haag, Naomi Rifaela, Gerrieke Beukema, Ron H. J. Mathijssen, Neeltje Steeghs, Hans Gelderblom, Ingrid M. E. Desar, Arjen Cleven, Arja ter Elst, Ed Schuuring, Anna K. L. Reyners

**Affiliations:** ^1^ Department of Medical Oncology and Pathology, University Medical Center Groningen University of Groningen The Netherlands; ^2^ Department of Medical Oncology, Erasmus MC Cancer Institute Erasmus University Medical Center Rotterdam The Netherlands; ^3^ Department of Medical Oncology The Netherlands Cancer Institute Antoni van Leeuwenhoek Amsterdam The Netherlands; ^4^ Department of Medical Oncology Leiden University Medical Center The Netherlands; ^5^ Department of Medical Oncology Radboud University Medical Center Nijmegen The Netherlands

**Keywords:** circulating tumor DNA, follow‐up, gastro‐intestinal stromal tumor, treatment, tumor volume

## Abstract

Patients with gastro‐intestinal stromal tumors (GISTs) undergoing tyrosine kinase inhibitor therapy are monitored with regular computed tomography (CT) scans, exposing patients to cumulative radiation. This exploratory study aimed to evaluate circulating tumor DNA (ctDNA) testing to monitor treatment response and compare changes in ctDNA levels with RECIST 1.1 and total tumor volume measurements. Between 2014 and 2021, six patients with KIT proto‐oncogene, receptor tyrosine kinase (*KIT*) exon‐11‐mutated GIST from whom long‐term plasma samples were collected prospectively were included in the study. ctDNA levels of relevant plasma samples were determined using the KIT exon 11 digital droplet PCR drop‐off assay. Tumor volume measurements were performed using a semi‐automated approach. In total, 94 of 130 clinically relevant ctDNA samples were analyzed. Upon successful treatment response, ctDNA became undetectable in all patients. At progressive disease, ctDNA was detectable in five out of six patients. Higher levels of ctDNA correlated with larger tumor volumes. Undetectable ctDNA at the time of progressive disease on imaging was consistent with lower tumor volumes compared to those with detectable ctDNA. In summary, ctDNA levels seem to correlate with total tumor volume at the time of progressive disease. Our exploratory study shows promise for including ctDNA testing in treatment follow‐up.

## Graphical abstract

Patients with gastro‐intestinal stromal tumors on tyrosine kinase inhibitor therapy are exposed to cumulative radiation from routine CT scans. ctDNA analysis is emerging as a non‐invasive approach to assess treatment response and disease progression. Elevated ctDNA levels seem to correlate with larger total tumor volumes at the time of progressive disease, indicating a correlation between ctDNA levels and total tumor volume.

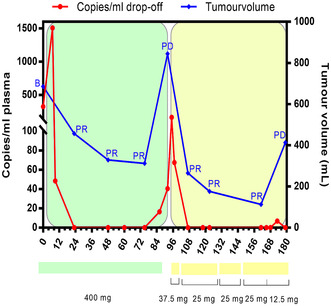

**Abbreviations**
cfDNA: circulating cell‐free DNA
CR: complete remission according to RECIST
CT: computed tomography
ctDNA: circulating tumor DNA
ddPCR: digital droplet polymerase‐chain‐reaction
FFPE: paraffin‐embedded
GIST: gastro‐intestinal stromal tumor
HU: hounsfield unit
IQR: interquartile range
KIT: KIT proto‐oncogene, receptor tyrosine kinase
NS: next‐generation sequencing
PD: progressive disease according to RECIST
PDGFRA: platelet‐derived growth factor receptor alpha
PR: partial response according to RECIST
RECIST: Response Evaluation Criteria In Solid Tumors
SD: stable disease according to RECIST
TKI: tyrosine kinase inhibitor
UMCG: University Medical Center of Groningen

## 1 Introduction

Gastro‐intestinal stromal tumors (GIST) are rare mesenchymal tumors arising in the gastro‐intestinal tract [1]. The most common locations are the stomach (50–60%) and the small bowel (20–30%) [2, 3]. GISTs are characterized by gain‐of‐function mutations in genes encoding for the tyrosine kinase receptors KIT proto‐oncogene, receptor tyrosine kinase (*KIT*) and platelet‐derived growth factor receptor alpha (*PDFGRA*). The majority of patients with GIST harbor a *KIT* gene mutation (70%), which most often affects *KIT* exon 11 (90%) [4].

In localized GISTs the primary treatment is surgery. However, 15–50% of the patients present with metastatic disease at diagnosis, initially to the liver or peritoneum [1, 5, 6, 7, 8]. Metastatic disease or locally advanced irresectable tumors are treated with tyrosine kinase inhibitors (TKIs) such as imatinib, sunitinib, regorafenib, and ripretinib [9, 10, 11, 12, 13, 14]. Response rates vary by primary mutation [15, 16]. Therefore, molecular diagnostic predictive testing on pretreatment tumor biopsies, using a variety of methods such as next‐generation sequencing (NGS), is recommended.

For optimal decision making, computed tomography (CT) scans are made every 3–6 months to monitor response during treatment with TKIs. Patients with metastatic GISTs can receive long‐term treatment with TKIs, with a median overall survival of over 57 months [14]. As a result of the intensive follow‐up schedule, patients can be exposed to significant accumulative levels of radiation. Worldwide, GIST lesions are mostly measured according to the Response Evaluation Criteria In Solid Tumors (RECIST) [17]. However, RECIST has its limitations since only a maximum of five lesions are identified as target lesions which does not always reflect total tumor burden in patients with metastasized GIST. Moreover, response cannot always be measured correctly using conventional criteria due to changes in tumor density and cyst formation without significant changes in tumor size [18]. Therefore, Choi et al [19]. developed a new method including changes in tumor vascularity after initiation of TKI treatment leading to a decreased tumor density which is reflected by the Hounsfield unit (HU) value on CT. However, Choi‐criteria seemed to gradually overestimate the number of patients with a response to TKI treatment during long‐term follow‐up because a decrease in tumor density could also be caused by tumor necrosis, indicating progressive disease [20]. Recently, measurement of total tumor volume (also called 3D) was evaluated as an alternative response measurement in patients with GIST treated with TKIs. These studies showed that total tumor volume measurement is more sensitive to detect a reduction in tumor size compared with RECIST (which is actually 1D) [21, 22].

Circulating tumor DNA (ctDNA) to monitor treatment response in patients with various malignancies has gained interest in recent years [23]. ctDNA is part of circulating cell‐free DNA (cfDNA), which refers to fragmented DNA molecules that are freely circulating in the bloodstream, derived from normal cells and cells involved with pathologic processes (including cell death) [24]. ctDNA analysis involves the quantification and detection of tumor‐derived DNA shedded into the bloodstream due to apoptosis and necrosis [25]. This technique seems to offer a faster, less invasive, cost‐effective and patient‐friendly alternative to CT scanning. Recent studies in other malignancies showed that ctDNA analysis provides a more accurate reflection of complete response compared to imaging measurements, while an increase in ctDNA is associated with poor prognosis [26, 27]. Therefore, the detection of changes in ctDNA levels offers the opportunity to evaluate treatment response and is referred to as molecular response.

Previously, the detection of both ctDNA at baseline and at early response of TKI treatment in patients with *KIT* exon‐11‐mutated GIST was studied by using a validated *KIT* exon 11 digital droplet polymerase‐chain‐reaction (ddPCR) drop‐off assay. These results showed that ctDNA could be detected at baseline in 13 of 14 patients with metastatic GIST. A decrease in ctDNA during systemic treatment was seen in all patients [29]. Additionally, more recent research demonstrated a *KIT* mutation detection rate of 75% in baseline (pretrial treatment) plasma samples of patients with advanced GIST who were previously treated with imatinib [30]. The potential to predict TKI activity based on ctDNA analysis was demonstrated since the detection of *KIT* mutations in plasma appeared to correlate with outcomes in patients with metastatic TKI‐resistant GIST treated with third‐ and fourth‐line TKI treatment. However, a meaningful clinical use remains debated since previous studies showed that ctDNA levels in plasma are low and there is a high variance between individual patients with GIST [28, 29, 31, 32].

Previous studies suggest that ctDNA reflects total tumor burden based on tumor volumes in patients with colorectal cancer [33]. Given the positive relation of ctDNA and tumor volume and to better understand the clinical utility of ctDNA, the present study evaluated ctDNA during the long treatment course of patients with GIST by using the *KIT* exon 11 ddPCR drop‐off assay [29] and compared changes in ctDNA levels with RECIST and total tumor volume measurements obtained from CT scans. The aim of this exploratory study was to assess the clinical utility of ctDNA to monitor long‐term treatment response in individual GIST patients, as well as the detection of progression by ctDNA.

## 2 Materials and methods

### 2.1 Study design

This study is part of a cohort multicenter study aiming to detect the value of primary and secondary GIST mutations in cfDNA of patients with progressive disease on TKI treatment. This study is registered on ClinicalTrials.gov (NCT02331914). All included patients gave written informed consent. The Medical Ethical Committee of the University Medical Center of Groningen (UMCG) approved the study (METc 2020/001). The study methodologies conformed to the standards set by the Declaration of Helsinki.

### 2.2 Study population

Between 2014 and 2021, we prospectively collected plasma samples from patients with GIST included in the overarching cfDNA trial NCT02331914. Criteria for the current analyses were locally advanced or metastatic *KIT* exon‐11‐mutated GIST, planned treatment with TKIs and treatment and follow‐up at the UMCG. All prospectively collected samples were screened on clinical relevance. Clinical relevant samples were defined as samples that were collected at the moment of response to treatment or progressive disease based on RECIST version 1.1, or after start of a new treatment line. Six patients of whom the primary *KIT* exon 11 mutation could be detected in cfDNA at diagnosis or at time of recurrent disease, before start of TKI treatment, with additional clinically relevant plasma samples at long‐term were selected out of the previously published trial (Fig. S1) patients.

### 2.3 Analysis of ctDNA by ddPCR

The level of ctDNA was measured using the in‐house designed and validated *KIT* exon 11 ddPCR drop‐off assay. This assay is able to detect 80% of all KIT exon 11 mutations that occur in one of the two hotspot regions (position c.1641‐c.1765). The assay consists of PCR primers and two TaqMan probes labeled with FAM and HEX fluorescent, respectively. The method of the detection of ctDNA using this assay has been published before [29]. In short, mutational status was previously determined by routine NGS in all patients. A ddPCR analysis according the Bio‐Rad procedure [34] using the *KIT* exon 11 drop‐off assay was first performed on paraffin‐embedded (FFPE) tissue, retrieved from pretreatment biopsies. For each ddPCR an input of 2 ng genomic DNA, measured by Qubit dsDNA High Sensitivity Assay kit of Invitrogen, was used. Next, a ddPCR analysis was performed on the selected prospectively collected plasma samples. Cell Free DNA Streck tubes or EDTA tubes were used to collect blood samples. For STRECK BCT tubes within 5 days and for EDTA tubes within 4 h after the blood sample was taken, plasma was processed as previously described [29, 35] and stored in aliquots of 1 mL at −80 °C. For analysis, samples were thawed and cell‐free DNA (cfDNA) was isolated using Qiagen's Circulating Nucleic Acid *KIT* (Qiagen, Hilden, Germany). The cfDNA concentrations were measured by the Qubit dsDNA High Sensitivity Assay kit. Samples are diluted to a concentration of 0.67 ng·μL^−1^. A total of 8.8 μL of the dilution is added to the ddPCR analysis, resulting in a total input of 5.9 ng cfDNA. Samples with lower cfDNA concentrations were not diluted and a minimum of 0.25 ng·L^−1^ must be reached for analysis. For ddPCR analysis, a total of 22 μL was used, including diluted cfDNA (8.8 μL) and ddPCR mastermix including primers and probes of the assay (13.2 μL). For every ddPCR analysis positive mutant hotspot 1, positive mutant hotspot 2, wild‐type (SiHa cell line) and NTC (H_2_O) controls were added. Using the QX200 Droplet generator, droplets were generated after adding 70 μL droplet generator oil (Bio‐Rad Laboratories, Pleasanton, CA, USA). In the QX200 Droplet Reader (Bio‐Rad Laboratories), FAM and HEX labeled droplets were measured using the quantasoft software version 1.6.6 (Bio‐Rad Laboratories). A sample was considered positive when 3 or more FAM or HEX positive droplets (mutant droplets) were detected in minimum of 303 droplets total (mutant and wild‐type droplets), resulting in a minimum sensitivity of 1%. In case of < 3 mutant droplets, a sample was considered negative which means that ctDNA was undetectable. The level of ctDNA was calculated in mutant copies per mL of plasma, based on the primary *KIT* exon 11 mutation. The mutant copies per μL of plasma were calculated by the Quantasoft software.

All analyses were performed in the ISO15189‐accredited laboratory of molecular pathology at the UMCG where all precautions are taken to prevent contamination. The laboratory technicians were not aware of the CT scan results (RECIST as well as tumor volumes) while performing the ddPCR analyses.

### 2.4 Tumor measurement

Response or follow‐up evaluations were conducted according to the national guidelines by using contrast enhanced CT scans. Treatment response was assessed by RECIST version 1.1 [17]. Volumetric 3D tumor measurements were performed by an independent radiologist, who was unaware of the measured ctDNA levels. Siemens Syngo.via software (Enterprise Browser VB30 HF06, VB40A HF02 and VB50 HF02) was used in a semi‐automated manner, allowing for manual correction to assess tumor volumes. All lesions with a maximal diameter > 10 mm were included for total volumetric measurements. Tumor volume measurements were performed exclusively on CT scans accompanied by a blood sample collected within a 3‐week period.

## 3 Results

### 3.1 Baseline characteristics

**Fig. 1:**
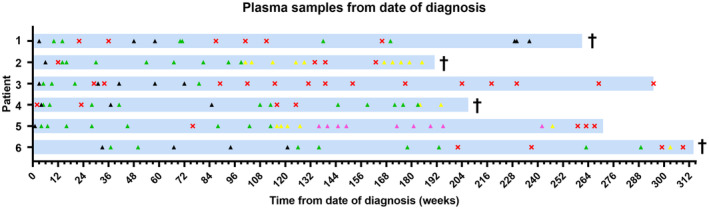
Selection of prospectively collected plasma samples. Time from diagnosis to last follow‐up (= light blue bar). Plasma samples that were not analyzed by KIT exon 11 digital droplet polymerase‐chain‐reaction (ddPCR) drop‐off assay (= red crosses). Clinically relevant plasma samples that were analyzed by ddPCR without treatment (= black triangles). Plasma samples that were analyzed by ddPCR under imatinib treatment (= green triangles), sunitinib treatment (= yellow triangles) or regorafenib treatment (= purple triangles), respectively. † = dead due to disease.

A total of 130 plasma samples were prospectively collected before and during treatment with TKIs. A total of 94 clinically relevant samples were analyzed (Fig. 1). The median time from diagnosis to last follow‐up was 266 (IQR 203–300) weeks. A median of 15 (IQR 11–20) samples were analyzed per patient.

### 3.2 RECIST versus tumor volume measurements

**Fig. 2:**
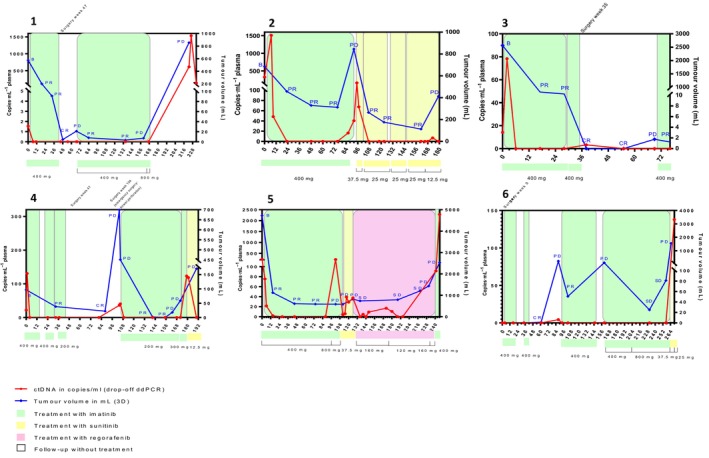
ctDNA versus tumor volume and RECIST. Figure shows the circulating tumor DNA (ctDNA) in copies·mL^−1^ (red line), analyzed by KIT exon 11 digital droplet polymerase chain reaction (ddPCR) drop‐off assay, in association with total 3D tumor volume in mL (blue line). All patients had metastasized disease at first time of detection of ctDNA. All blood samples in which ctDNA was measured were taken within 3 weeks of imaging. The *x*‐axis shows time in weeks from first taken blood sample. The left *y*‐axis shows the scale of copies·mL^−1^ and the right *y*‐axis of total 3D tumor volume (mL). The colors in the background correspond to the different treatment lines. Response evaluations according to Response Evaluation Criteria In Solid Tumors (RECIST 1.1): B, baseline; CR, complete response; PD, progressive disease; PR, partial response; SD, stable disease.

In 3 patients recurrence was detected earlier in follow‐up by measurements of 3D tumor volume compared with RECIST. In patient 1 at week 48, a tumor volume of 4.4 mL was measured in the radiofrequency ablation cavity. In patient 4 and 6, tumor volumes of 22.3 mL and 1.5 mL were measured at week 88 and week 61, respectively. In patient 6, stable disease according to RECIST was seen at week 257, while the tumor volume increased from 24.9 mL (week 231) to 81.2 mL (week 257; Fig. 2 and Table S1).

Patient 5 showed progressive disease (PD) based on RECIST at week 102, while tumor volume remained stable from the previous measurement (588 and 587.1 mL, respectively; Fig. 2). PD was based on new lesions in de liver with diameters of 37 and 23 mm, respectively.

### 3.3 ctDNA in relation to radiological response

In all six patients of whom partial response or stable disease according to RECIST was seen on imaging, levels of ctDNA became undetectable. When tumor volumes decreased over time, ctDNA was immediately undetectable at time of (or even before) the first tumor volume measurement after initiation of therapy in five patients, even when tumor volumes ranging from 0.50 to 456.87 mL were measured. A more gradual decrease of the level of ctDNA [225.25–75.34–21.44–1.92 copies·mL^−1^ (Fig. 2, Table S1) was seen in patient 5]. A low amount of ctDNA (1.92 copies·mL^−1^) was still detectable at the time of initial tumor volume decrease. At that time a high tumor volume (1122.36 mL) was measured.

In 4 patients with a temporary period of complete remission, ctDNA levels were undetectable in all 8 follow‐up samples. Except for one sample in patient 3 (Fig. 2) that was taken 2 weeks after surgery. In that sample a ctDNA level of 3.3 mutant copies·mL^−1^ could be detected while there was no evidence of disease.

All six patients had one or more episodes of PD on imaging. In five patients PD could be detected by ctDNA at least once. The level of ctDNA at the time of progressive disease varied from 4.2 to 2531 copies·mL^−1^ (Fig. 2, Table S1). ctDNA could not be detected in 9 out of 15 samples taken at the time of PD. All patients of whom an increase of tumor volume was measured with undetectable ctDNA levels, showed lower tumor volumes compared with the tumor volumes measured in which ctDNA could be detected. For example, in patient 1 (Fig. 2, Table S1) at week 67 and week 160, increasing tumor volumes of 21.2 mL and 7.2 mL could not be detected by an increase in ctDNA levels. These tumor volumes were lower compared to the tumor volume at week 221 (855.2 mL) in which the primary *KIT* exon 11 mutation could be detected in cfDNA. Also in patient 3 the primary *KIT* exon 11 mutation could not be detected in cfDNA while the tumor volume increased from 0 to 1.75 mL at the time of PD (recurrence). The *KIT* exon 11 mutation was detectable in cfDNA at baseline, when the tumor volume was 2563.3 mL. This was similar for patient 6, at week 160, with an increasing tumor volume of 100.4 mL, ctDNA could not be detected. Previously at week 88, showing a doubled tumor volume of 218.8 mL, ctDNA could be detected.

In patient 2, an increase of ctDNA in both week 86 and 173 was detected 6 weeks before PD on imaging was detected (after previously reported response to treatment; Fig. 2, Table S1).

**Fig. 3:**
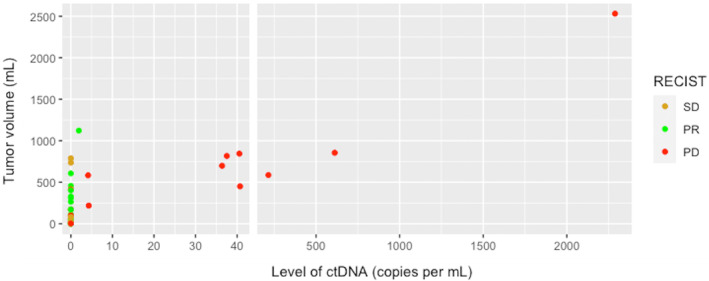
Tumor volume versus RECIST and ctDNA. Figure shows the relation of circulating tumor DNA (ctDNA) in copies·mL^−1^ with total 3D tumor volume in mL of all samples of all patients that were taken at time of PR (green), SD (brown) or PD (red). SD = stable disease (*n* = 4), PR = partial response (*n* = 14), PD = progressive disease (*n* = 15), according to Response Evaluation Criteria In Solid Tumors (RECIST 1.1). Only blood samples taken within 3 weeks of imaging were included. Samples at time of complete response (CR) according to RECIST were excluded (*n* = 6).

Figure 3 shows that at PD according to RECIST, higher tumor volumes (above 450 mL) correlate with higher levels of ctDNA. In case of partial response or stable disease, higher tumor volumes do not result in detectable ctDNA, unless the tumor volume was above 1000 mL.

### 3.4 Course of ctDNA during systemic treatment

An increase in the level of ctDNA (so‐called ‘ctDNA spike’) was found in four patients (patients 2–4, 6), shortly (within 2–3 weeks) after start of treatment. In patient 4 and 5 (Fig. 2), a ctDNA spike was seen after start of all treatment lines. During the follow‐up of patient 5, several ctDNA spikes were seen, which could be associated with this intermitted dosing schedule of regorafenib (3 weeks on 1 week off). ctDNA spikes after initiation of imatinib preceded imaging responses, while ctDNA spikes after sunitinib did not always correlate with imaging responses (Fig. 2; patient 4 and 5).

## 4 Discussion

This study demonstrated the clinical utility of ctDNA in monitoring TKI treatment response in patients with *KIT* exon‐11‐mutated GIST. Our findings demonstrate a strong correlation between ctDNA levels and tumor volume, supporting the utility of ctDNA testing. Especially at the time of progressive disease (PD), tumor volume seems to reflect ctDNA levels better than RECIST 1.1.

Whilst PD based on RECIST could not always be detected by ctDNA, this study showed that the increase of tumor volume more accurately correlates with an expected increase in ctDNA. In patients with evidence of GIST shedding DNA into the bloodstream, higher tumor volumes (> 450 mL) during PD according to RECIST more often showed higher levels of ctDNA. In each individual patient with PD on imaging, undetectable ctDNA was consistent with lower tumor volumes than detectable ctDNA.

In patient 2, early detection of PD was seen in ctDNA levels. Similar patterns were possibly seen in patient 4 (week 179 and 181) and patient 5 (week 241). However, in these patients, this increase of ctDNA could not be distinguished from a ctDNA spike after initiation of a new therapy or as a result of previously detected PD on imaging. Nevertheless, these results might show the potential use of ctDNA as an early marker for PD in patients with GIST, which also has previously been described in non–small‐cell lung cancer [36].

Tumor volume measurement seemed to detect recurrence earlier than RECIST. Moreover, patient 6 showed an incidental increase in tumor volume while stable disease according to RECIST was previously seen, which could have been an early detection of PD. However, we should note that the radiologist may have looked at the images in more detail given the study context. One patient (patient 5) showed PD according to RECIST based on 2 new liver metastasis, while the total tumor volume slightly decreased. However, this patient had over 100 liver and peritoneal metastases, which limits the use of only 2 target lesions in the liver to monitor treatment response.

The tumor volume threshold in which ctDNA could be detected varied per patient. Other reasons for unexpected low ctDNA levels might be inter‐ and intratumor heterogeneity of GIST and the development of secondary (resistant) mutations during treatment which are spatially distributed in the primary tumor [37]. This may result in elevated ctDNA levels that are undetectable for the *KIT* exon 11 ddPCR drop‐off assay, despite evidence of PD on imaging. Nonetheless, recent research showed that the majority of patients with advanced *KIT* mutated GIST with resistance to treatment with imatinib had 1–3 *KIT* mutations and only 17% showed substantial heterogeneity with 4–14 *KIT* mutations detected in plasma [30].

In case of partial response and stable disease according to RECIST, the level of ctDNA became undetectable in all patients, except for one. In that patient, a gradual decrease of the ctDNA level was seen. This could be explained by high tumor volumes at baseline that decreased during treatment. Only in that patient, tumor volume correlated better with ctDNA than partial response based on RECIST.

Interestingly, an early molecular response was seen in most patients, with a rapid decrease in ctDNA around 3 weeks after initiation of systemic therapy, before the first radiological response. CtDNA spikes were detected within 2 weeks after starting systemic treatment, prior to a vanishing level of ctDNA in patients who responded to treatment with imatinib and sunitinib. These spikes do not reflect radiological response but can be explained by acute response at start of systemic treatment inducing apoptosis and necrosis resulting in higher release of ctDNA into the bloodstream [25]. Due to the large amount of DNA that is released after initiation of therapy, it must also be taken into account in what values (fractional abundance (FA) or mutant copies per mL) the level of ctDNA is measured. CtDNA also contains large amounts of wild‐type DNA [emanating from other (benign) cells without the primary *KIT* exon 11 mutation]. Therefore, ctDNA levels expressed in FA, which is calculated as the ratio between the mutant and wild‐type DNA, can give a distorted perspective. For that reason, in this study, we used mutant copies per mL as measurement for ctDNA levels. After 3 weeks of treatment, the ctDNA spikes had diminished completely, which highlights the previously reported short half life time of ctDNA of 15 min up to 2,5 h [38]. Therefore, it seems to be important to further define the right timing of blood sampling to detect ctDNA. Multiple factors such as different dosing patterns also seem to influence the levels of ctDNA. For example, patient 6 showed a remarkable variable pattern of the level of ctDNA during intermitted dosing patterns in the treatment with regorafenib. Our study also indicates a possible link with surgery and the (short‐term) release of ctDNA into the bloodstream as low levels of detectable ctDNA were found in patient 3, 2 weeks after surgery. This patient developed a recurrence 6 months later, which also suggests that ctDNA could be a possible prognostic indicator for residual disease, as previously published in patients with colorectal cancer [39].

This study was the first to show a correlation of tumor volume with the level of ctDNA in metastasized GIST patients during long‐term treatment with different TKI treatment lines. Despite its small sample size, it gives us valuable starting points for future research. Regardless of introducing selection bias by selecting only six patients from a larger cohort, we avoided cherry‐picking by analyzing all 94 samples from these six patients. Larger sample sizes are needed to further investigate the clinical utility of ctDNA in GIST patients as new follow‐up tool to monitor response, especially in relation to tumor volume. Besides the fact that 30% of GIST possibly do not shed ctDNA [40], further investigation of prognostic factors influencing the level of ctDNA is needed. The impact of secondary mutations, different dosing patterns (including drug monitoring) and a better understanding of the time‐dependent release of ctDNA into the bloodstream should be studied to clarify the varying patterns of ctDNA in individual GIST patients. In time, plasma ctDNA could prove to be a reliable, cheaper, safer (reduced radiation) and more effective alternative to monitor GIST treatment with regard to CT‐imaging.

## 5 Conclusion

Our findings show a strong correlation between ctDNA levels and tumor volume. CtDNA became undetectable upon successful treatment response, while its presence was observed in patients with high tumor volumes at time of progressive disease. These results suggest that ctDNA analysis can provide valuable insights into treatment efficacy and disease progression, offering a non‐invasive and potentially more efficient approach compared to regular CT scans.

## Conflict of interest

Ed Schuuring has received unrestricted grants (all paid to UMCG institution) from Abbott, Biocartis, AstraZeneca, Invitae/Archer, Bayer, Bio‐Rad, Roche, Agena Bioscience, CC Diagnostics, MSD/MERCK, and Boehringer Ingelheim, has received consulting fees (all paid to UMCG institution) from MSD/Merck, AstraZeneca, Roche, Novartis, Bayer, BMS, Lilly, Amgen, Illumina, Agena Bioscience, CC Diagnostics, Janssen Cilag (Johnson & Johnson), Astellas Pharma, GSK, Sinnovisionlab, and Sysmex, has received payments or honoraria (all paid to UMCG institution) from Bio‐Rad, Seracare, Roche, Biocartis, Lilly, Agena Bioscience, and Illumina, has received support for attending meetings and/or travel from Bio‐Rad, Biocartis, Ageno SciencAR, and Illumina, is a board member for the Dutch Society of Pathology (unpaid), European Society of Pathology (unpaid), European Liquid Biopsy Society (unpaid), is a secretary/member of the advisory committee for assessment of molecular diagnostics (cieBOD) (honoraria paid to UMCG institution), is committee member of national guideline advisory (honoraria paid to UMCG institution). Anna K. Reyners has received payments for studies (all paid to UMCG institution) from Deciphera, GSK, MSD, Genmab, Cogent, Tesaro, and Regeneron. Anna K. Reyners is chairperson of the Committee for the Evaluation of Oncological Agents of the Dutch Society of Medical Oncologists (NVMO) and member of the board of the NVMO (paid to the UMCG institution). N Steeghs provided consultation or attended advisory boards for Boehringer Ingelheim, Ellipses Pharma, GlaxoSmithKline, Incyte, Luszana. N Steeghs received research grants from Abbvie, Actuate Therapeutics, Amgen, Array, Ascendis Pharma, AstraZeneca, Bayer, Blueprint Medicines, Boehringer Ingelheim, Bristol‐Myers Squibb, Cantargia, CellCentric, Cogent Biosciences, Cresecendo Biologics, Cytovation, Deciphera, Dragonfly, Eli Lilly, Exelixis, Genentech, GlaxoSmithKline, IDRx, Immunocore, Incyte, InteRNA, Janssen, Kinnate Biopharma, Kling Biotherapeutics, Lixte, Luszana, Merck, Merck Sharp & Dohme, Merus, Molecular Partners, Navire Pharma, Novartis, Numab Therapeutics, Pfizer, Relay Pharmaceuticals, Revolution Medicin, Roche, Sanofi, Seattle Genetics, Taiho, Takeda. All outside the submitted work, all payment to the Netherlands Cancer Institute. Ingrid M.E. Desar received payments for studies (all paid to Radboud University Medical Centre) from Sanofi‐Regeneron. She received a grant of the Dutch Cancer Foundation ‘Koningin Wilhemina Fund’, paid to Radboudumc. She is member of the Dutch Health Care Council, also paid to Radboudumc. Ron H.J. Mathijssen received unrestricted grants (all paid to the institute) for investigator initiated studies from Astellas, Bayer, Boehringer Ingelheim, Cristal Therapeutics, Novartis, Pamgene, Pfizer, Roche, Sanofi and Servier. The other authors declare no conflicts of interest.

## Author contributions

RFB: Conceptualization, Methodology, Formal analysis, Investigation, Data Curation, Writing—Original Draft. CMSCH: Formal analysis, Data Curation, Writing—Review & Editing. NR: Formal analysis, Data Curation, Writing—Review & Editing. GB: Formal analysis, Data Curation, Writing—Review & Editing. RHJM: Data Curation, Writing—Review & Editing. NS: Data Curation, Writing—Review & Editing. HG: Data Curation, Writing—Review & Editing. IMED: Data Curation, Writing—Review & Editing. AC: Data Curation, Writing—Review & Editing. AE: Methodology, Validation, Formal analysis, Writing—Review & Editing, Supervision. ES: Conceptualization, Methodology, Validation, Formal analysis, Writing—Review & Editing, Supervision. AKLR: Conceptualization, Methodology, Validation, Formal analysis, Writing—Review & Editing, Supervision.

### Peer review

The peer review history for this article is available at https://www.webofscience.com/api/gateway/wos/peer‐review/10.1002/1878‐0261.13644.

## Supporting information


**Fig. S1.** Flowchart patient selection.
**Table S1.** Supplementary information associated with Fig. 2: detailed overview of ctDNA analysis, tumor volume measurements, RECIST outcomes and treatment strategies of all patients.

## Data Availability

Data sharing is not applicable to this article as no new data were created or analyzed in this study.
