# Supplementary material for: Levels of circulating tumor DNA correlate with tumor volume in gastro‐intestinal stromal tumors: an exploratory long‐term follow‐up study
Source: Mol Oncol. 2024 May 24;18(11):2658–67. doi: 10.1002/1878-0261.13644 (PMC11547224; doi:10.1002/1878-0261.13644)
Supplement: Supplementary file 1 — Fig. S1. Flowchart patient selection. Table S1. Supplementary information associated with Fig. 2: detailed overview of ctDNA analysis, tumor volume measurements, RECIST outcomes and treatment strategies of all patients. [file MOL2-18-2658-s001.pdf]

Figure S1. Flowchart patient selection.

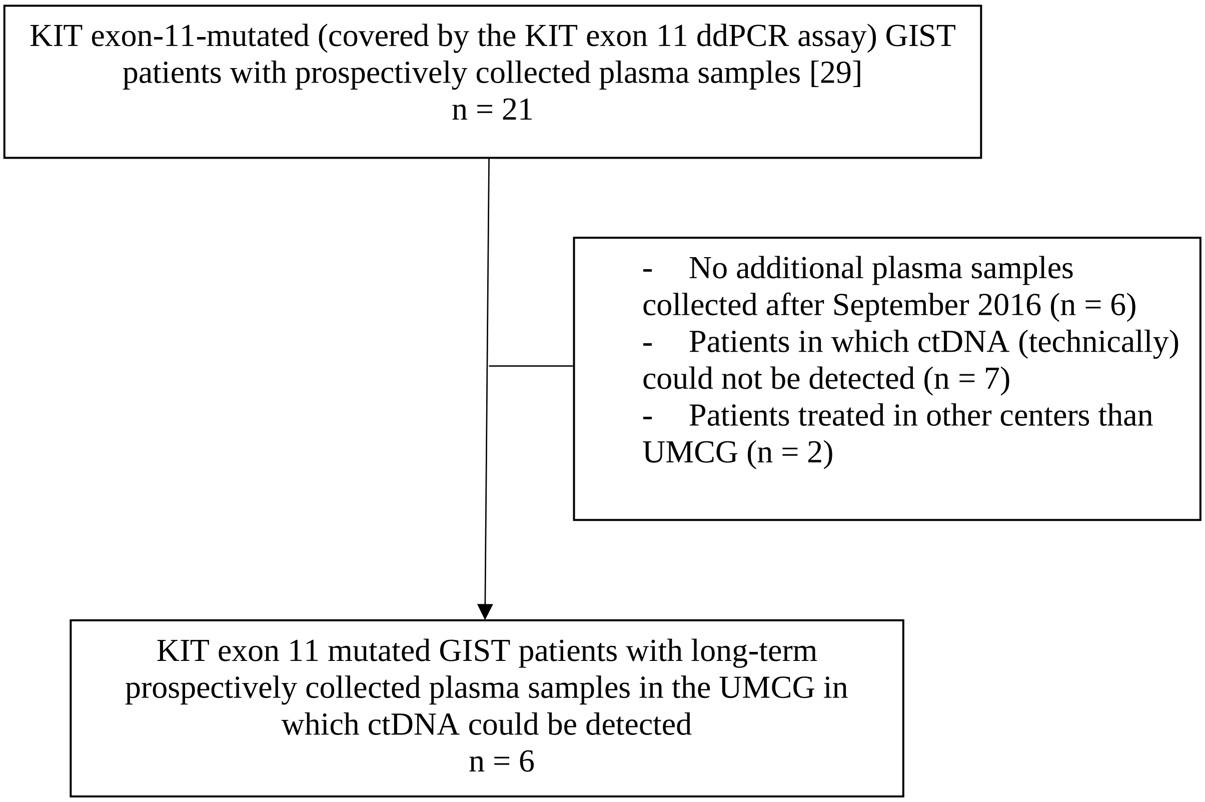

KIT = KIT proto-oncogene, receptor tyrosine kinase, ddPCR = digital droplet polymerase-chain-reaction, GIST = gastro-intestinal stromal tumor, ctDNA = circulating tumor DNA, UMCG = University Medical Center of Groningen.

Table S1. Supplementary information associated with Figure 2. Detailed overview of ctDNA analysis, tumor volume measurements, RECIST outcomes and treatment strategies of all patients.

Patient	Weeks from first taken plasma*	Weeks after start of new treatment or dose	Treatment	RECIST	Tumor volume (mL)	Level of ctDNA (copies per mL)	Total amount of droplets (ddPCR)	Total amount of mutant droplets (ddPCR)	Sensitivity
1	0		None	Baseline	576.11	1.54	4472	4	0.07
	7	3	Imatinib 400 mg			0	412	2	0.73
	11	7	Imatinib 400 mg			0	471	0	0.64
	19	13	Imatinib 400 mg	PR	200.69				
	33	28	Imatinib 400 mg	PR	90.98				
	38	34	Imatinib 400 mg	PR					
	45	41	None			0	1541	1	0.19
	47		Surgery						
	48		None	CR	4.4				
	55		None			0	1999	1	0.15
	67		None	PD	21.16	0	554	0	0.54
	68	1	Imatinib 400 mg			0	1716	1	0.17
	84	15	Imatinib 400 mg	PR	7.88				
	106	39	Imatinib 400 mg	PR					
	135	68	Imatinib 400 mg	PR	3.35	0	730	0	0.41
	160	93	Imatinib 400 mg	PD	7.20				
	167	2	Imatinib 800 mg			0	634	0	0.47
	223		None	PD	855.17	612.04	849	134	0.35
	226		None			1534.34	963	155	0.31
	233		None			132.82	1175	25	0.26
2	0			Baseline	685.54	323.67	1086	166	0.28
	7	2	Imatinib 400 mg			1506.84	1938	1195	0.15
	9	3	Imatinib 400 mg			48.43	635	22	0.47
	23	17	Imatinib 400 mg	PR	456.87	0	712	2	0.42
	48	42	Imatinib 400 mg	PR	329.14	0	310	0	0.97
	60	54	Imatinib 400 mg			0	304	0	0.99
	75	69	Imatinib 400 mg	PR	312.2	0	448	0	0.67
	86	80	Imatinib 400 mg			16.43	2825	61	0.11
	92	86	Imatinib 400 mg	PD	844.62	40.54	1397	58	0.21
	95	1	Sunitinib 37.5 mg			162.28	4945	201	0.06
	97	4	Sunitinib 37.5 mg			67.5	1971	47	0.15
	107	5	Sunitinib 25 mg	PR	265.25	0	2023	0	0.15
	118	16	Sunitinib 25 mg			0	720	0	0.42
	123	20	Sunitinib 25 mg	PR	176.45	0	751	1	0.40
	142	40	Sunitinib 25 mg	PR					
	161	58	Sunitinib 25 mg	PR	113.07	0	1039	0	0.29
	165	62	Sunitinib 25 mg			0	791	0	0.38
	168	4	Sunitinib 12.5 mg			0	364	0	0.82
	173	8	Sunitinib 12.5 mg			6.8	468	3	0.64
	179	14	Sunitinib 12.5 mg	PD	413.22	0	752	2	0.40
3	0		None	Baseline	2563.29	14.17	1078	8	0.28
	2	2	Imatinib 400 mg			78.39	536	30	0.56
	6	6	Imatinib 400 mg			0	325	0	0.92
	17	17	Imatinib 400 mg	PR	402.25	0	1091	0	0.27
	28		None**	PR	169.73	0	1022	1	0.29
	35		Surgery						
	38		None	CR	0	3.3	1542	4	0.19
	55		None	CR	0	0	543	0	0.55
	69		None	PD	1.75	0	716	2	0.42
	76	6	Imatinib 400 mg			0	578	1	0.52
	84	14	Imatinib 400 mg	PR	0.66				
4	0			Baseline	96.67	22.23	2603	34	0.12
	1	1	Imatinib 400 mg			130.65	706	49	0.42

	4	4	Imatinib 400 mg			0	565	0	0.53
	15	15	Imatinib 400 mg	PR					
	24	1	Imatinib 400 mg**			0	1852	0	0.16
	33		None**	PR	38.38	0	1334	0	0.22
	37	4	Imatinib 200 mg**			0	1194	0	0.25
	47		Surgery						
	60		None	CR					
	81		None			0	671	0	0.45
	88		None	CR	22.3				
	104		Surgery	PD	698.78	36.38	3201	43	0.09
	105		None	PD	450.77	40.7	4384	51	0.006
	109	3	Imatinib 200 mg			0	2880	1	0.10
	116	10	Imatinib 200 mg	PR					
	126	20	Imatinib 200 mg	PR					
	141	35	Imatinib 200 mg	PR	2.14	0	755	1	0.40
	155	49	Imatinib 200 mg	PR	0.5	0	774	0	0.39
	163	56	Imatinib 200 mg	PD	18.01				
	168	62	Imatinib 200 mg			0	1189	0	0.25
	172	66	Imatinib 200 mg	PD	58.83	0	1213	0	0.25
	179	7	Imatinib 300 mg			123.19	516	30	0.58
	181	1	Sunitinib 12.5 mg			118.62	2577	128	0.12
	190	10	Sunitinib 12.5 mg	PD	170.45	0	1088	0	0.28
5	0			Baseline	4722.78	225.55	3390	267	0.09
	4	1	Imatinib 400 mg			75.34	1529	115	0.20
	6	3	Imatinib 400 mg			21.44	1674	46	0.18
	15	12	Imatinib 400 mg	PR	1122.36	1.92	1108	3	0.27
	28	25	Imatinib 400 mg			0	445	0	0.67
	45	42	Imatinib 400 mg	PR	606.72	0	569	0	0.53
	74	71	Imatinib 400 mg	PR	587.97				
	88	85	Imatinib 400 mg			0	970	0	0.31
	102	99	Imatinib 400 mg	PD	587.09	215.6	479	93	0.63
	112	10	Imatinib 800 mg	PD	583.86	4.15	608	5	0.49
	115	2	Sunitinib 37.5 mg			5.8	1113	4	0.27
	117	4	Sunitinib 37.5 mg			39.34	585	33	0.51
	120	7	Sunitinib 37.5 mg			29.1	530	17	0.57
	126	13	Sunitinib 37.5 mg	PD	817.17	37.52	568	30	0.53
	136	8	Regorafenib 160 mg	SD	736.54	0	900	0	0.33
	140	12	Regorafenib 160 mg			4.73	1211	3	0.25
	144	17	Regorafenib 160 mg			0	532	0	0.56
	148	21	Regorafenib 160 mg			9.28	1328	5	0.23
	151	24	Regorafenib 160 mg	SD					
	163	36	Regorafenib 160 mg	SD					
	172	45	Regorafenib 160 mg	SD		16.96	1122	15	0.27
	175	48	Regorafenib 160 mg	SD					
	180	4	Regorafenib 120 mg			10.38	826	6	0.36
	188	12	Regorafenib 120 mg	SD	788.57	0	667	2	0.45
	195	19	Regorafenib 120 mg			0	1321	1	0.23
	219	43	Regorafenib 120 mg	PD	1206.12				
	231	55	Regorafenib 120 mg	PD	1432.85				
	241	9	Regorafenib 160 mg			91.3	1510	42	0.20
	246	5	Imatinib 400 mg	PD	2530.68	2287.81	1605	452	0.19
6	0		None			0	3015	0	0.10
	4		None	CR	0	0	969	0	0.30

	17	13	Imatinib 400 mg			0	321	0	0.93
	34		None	CR	0	0	877	0	0.34
	61		None	CR	1.46	0	812	0	0.37
	88		None	PD	218.81	4.3	1287	5	0.23
	94	3	Imatinib 400 mg			0	398	0	0.75
	103	13	Imatinib 400 mg	PR	50.8	0	975	0	0.3
	116	26	Imatinib 400 mg	PR					
	132	41	Imatinib 400 mg	PR					
	145	55	Imatinib 400 mg			0	1421	2	0.21
	160	1	Imatinib 400 mg****	PD	100.44	0	1619	1	0.18
	168	8	Imatinib 400 mg	PR					
	183	23	Imatinib 400 mg	PR					
	204	44	Imatinib 400 mg	PD*****					
	213	9	Imatinib 800 mg	SD					
	231	26	Imatinib 800 mg	SD	24.89	0	1686	2	0.18
	257	52	Imatinib 800 mg	SD	81.17	0	679	1	0.44
	265	61	Imatinib 800 mg	PD	1546.25				
	270	5	Sunitinib 25 mg			137.84	1379	74	0.22

ctDNA = circulating tumor DNA, ddPCR = digital droplet polymerase-chain-reaction, CR = complete remission, SD = stable disease, PR = partial response, PD = progressive disease. Tumor volume measurements were done only for CT-scans where a blood sample had been taken within 3 weeks.

*Weeks from time of first taken blood sample in study. In case of level of ctDNA in combination with tumor volume, weeks are based on time of taken blood sample and imaging was performed within 3 weeks of taken blood sample.

** Multiple (temporary) interruptions of therapy due to adverse events.

*** Recurrence of disease after surgery and 3 years adjuvant imatinib (no evidence of disease).

**** Given the patient's wish 2 months before blood sample was taken no treatment. (4 days after restart treatment plasma sample was taken).

***** Blood sample was taken simultaneously, but insufficient material for analysis.
